# Risk factors for the onset of dependence and chronic psychosis due to cannabis use: Survey of patients with cannabis‐related psychiatric disorders

**DOI:** 10.1002/npr2.12133

**Published:** 2020-09-07

**Authors:** Toshihiko Matsumoto, Toshitaka Kawabata, Kyoji Okita, Yuko Tanibuchi, Daisuke Funada, Maki Murakami, Takashi Usami, Rie Yokoyama, Nobuya Naruse, Yuzo Aikawa, Aizo Furukawa, Chie Komatsuzaki, Nozomu Hashimoto, Osamu Fujita, Aiko Umemoto, Ariyuki Kagaya, Takuya Shimane

**Affiliations:** ^1^ Department of Drug Dependence Research National Center of Neurology and Psychiatry National Institute of Mental Health Kodaira Japan; ^2^ Center Hospital National Center of Neurology and Psychiatry Kodaira Japan; ^3^ Department of Psychiatry Kyoto Prefectural Rakunan Hospital Uji Japan; ^4^ Department of Clinical Neuroimaging Integrative Brain Imaging Center National Center of Neurology and Psychiatry Kodaira Japan; ^5^ Department of Psychiatry Kaisei Hospital Kasama Japan; ^6^ Department of Psychiatry Saitama Prefectural Psychiatric Hospital Saitama Japan; ^7^ Department of Psychiatry Seimei Hospital Fukuoka Japan; ^8^ Department of Psychiatry Ibaraki Prefectural Medical Center of Psychiatry Kasama Japan; ^9^ Department of Psychiatry Okayama Psychiatric Medical Center Okayama Japan; ^10^ Department of Psychiatry Osaka Psychiatric Medical Center Osaka Japan; ^11^ KONUMA Memorial Institute of Addiction and Mental Health Senogawa Hospital Hiroshima Japan

**Keywords:** cannabis, chronic psychosis, dependence, risk factor, use disorder

## Abstract

**Aim:**

The objective of the current study was to identify risk factors that affect the onset of dependence and chronic psychosis due to cannabis use.

**Methods:**

We examined clinical genetic factors, psychiatric disorders prior to cannabis use, starting age of cannabis use, duration and frequency of cannabis use, types of cannabis products used, combined use of other psychoactive substances, and the psychiatric diagnosis of 71 patients with cannabis‐related psychiatric disorders who underwent treatment at nine mental health hospitals in Japan. Information was collected from cross‐sectional interview surveys conducted by each patient's attending psychiatrist.

**Results:**

For the diagnosis of dependence syndrome due to the use of cannabis, we found associations with the number of years of cannabis use and the use of cannabis products with a high Δ9‐tetrahydrocannabinol (THC) content. However, we found no association between diagnosis of residual and late‐onset psychotic disorders and clinical genetic factors, presence of preceding psychiatric disorders, duration and frequency of cannabis use, starting age of cannabis use, or combined use of other psychoactive substances; an association was found only for the absence of use of cannabis products other than dried cannabis.

**Conclusion:**

The onset of cannabis dependence was related to long‐term cannabis use and the use of cannabis products with a high THC content. However, chronic psychosis was not associated with total THC intake or psychiatric vulnerability. Thus, unknown factors appear to be involved in the onset of chronic psychosis.

## Abstract 2

The onset of cannabis dependence was related to long‐term cannabis use and the use of cannabis products with a high THC content. However, chronic psychosis was not associated with total THC intake or psychiatric vulnerability.

## Graphical abstract

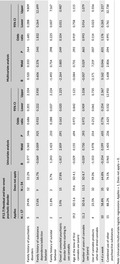

## 1 INTRODUCTION

Major changes in cannabis policies have recently occurred in a number of countries worldwide. For example, the Canadian government legalized cannabis for recreational use in October 2018.^1^ In the United States, cannabis for recreational use has been legalized in eight states, and the use of cannabis for medical purposes is already legal in 25 states,^2^ although the federal government does not tolerate cannabis use. These international trends are expected to have an impact on Japanese cannabis policies in the near future. Therefore, the health problems resulting from cannabis use, including the onset of dependence and psychiatric illness, are an important public health concern.

Although animal and human research has confirmed that cannabis is an addictive drug,^3^ only a limited number of people who use cannabis become addicted.^4^ A large‐scale epidemiological study conducted in Europe^5^ reported that the proportion of people who use cannabis and become addicted was significantly lower than the number of individuals who become addicted to nicotine, alcohol, or cocaine after use. From the perspective of dependence prevention, it is important to elucidate the risk factors for the onset of dependence due to cannabis use. In previous studies conducted in various countries, the frequency and duration of cannabis use, the Δ9‐tetrahydrocannabinol (THC) content of the cannabis product used,^6^, ^7^, ^8^ family history of substance dependence,^9^, ^10^, ^11^ presence of psychological distress before starting to use cannabis,^12^ and cannabis use early in life^11^, ^13^ were identified as risk factors for the onset of cannabis dependence.

Moreover, chronic psychosis, a psychiatric illness that can be difficult to differentiate from schizophrenia, is reported to be related to cannabis use. This association was reported for the first time by Andreasson et al in a longitudinal study of Swedish conscripts in the late 1980s.^14^ Since then, numerous studies have reported similar findings,^14^, ^15^, ^16^, ^17^, ^18^, ^19^, ^20^, ^21^, ^22^, ^23^, ^24^, ^25^ and it has been estimated that 6.2% to 24% of all psychiatric illnesses would not have occurred if the patient had not used cannabis.^26^ However, the number of people who use cannabis and develop a psychiatric illness is very small. Further, a study in the United Kingdom did not support a relationship between cannabis use and chronic psychosis, reporting that the national prevalence of schizophrenia remained unchanged or slightly decreased over a period in which the proportion of people who used cannabis increased.^27^ To date, identified risk factors for the onset of psychiatric illness related to cannabis use include a family history of psychiatric disorders,^28^, ^29^ cannabis use early in life,^13^ use of cannabis products with a high THC content,^30^ and the use of cannabis to alleviate the symptoms of psychiatric disorders.^31^

Whether these international findings apply to Japan, which has unique laws, regulations, and a national lifetime prevalence of cannabis use, is unclear. Only a small number of previous studies have examined cannabis‐related psychiatric disorders in Japan, and all of these studies were case reports with a maximum of six cases.^32^, ^33^, ^34^, ^35^, ^36^ Furthermore, these studies all focused on describing the clinical presentation of chronic psychosis based on the clinical concept of “amotivational syndrome,” which McGlothlins and West^37^ proposed as an after effect of cannabis use. No previous studies have examined risk factors for the onset of psychiatric illness and cannabis dependence in Japan.

Therefore, the current study was conducted to elucidate the risk factors that affect the onset of dependence and chronic psychosis due to cannabis use in a relatively large number of patients with cannabis‐related disorders. Moreover, we investigated the risk factors identified in previous studies, including clinical genetic factors, the impact of psychiatric disorders present prior to cannabis use, the starting age of cannabis use, duration and frequency of cannabis use, cannabis products (THC content) used, and the impact of the combined use of other psychoactive substances.

## 2 METHODS

### 2.1 Subjects

We selected nine mental health hospitals with more than 100 reported cases as study sites (Ibaraki Prefectural Medical Center of Psychiatry, Saitama Prefectural Psychiatric Hospital, National Center of Neurology and Psychiatry, Seimei Hospital, Kyoto Prefectural Rakunan Hospital, Osaka Psychiatric Medical Center, Okayama Psychiatric Medical Center, Senogawa Hospital, and Kaisei Hospital) among the 1264 mental health hospitals nationwide that participated in the 2018 Nationwide Mental Hospital Survey on Drug‐Related Psychiatric Disorders (hereinafter referred to as the “NMH Survey”)^38^. In the NMH Survey, these nine mental health hospitals reported 1328 cases of cannabinoid‐related psychiatric disorders, which accounted for approximately half (48%) of all 2767 cases of drug‐related psychiatric disorders. Each of the facilities had a specialized treatment system for drug dependence.

The patients were all adults who (a) fell under the ICD‐10 classification “F12—Mental and behavioral disorders due to use of cannabinoids,” (b) underwent treatment as an outpatient or inpatient at one of the nine psychiatric hospitals during the 3‐month period from October to December 2019, and (c) provided consent to participate in this study.

### 2.2 Survey procedure

For information collection, we employed a cross‐sectional method, using interviews conducted by the attending psychiatrists at the study sites. During therapy sessions, each attending psychiatrist directly asked patients who met the inclusion criteria questions after they had given verbal consent. Patient responses were entered into the survey sheet after judgment by the attending psychiatrists, who referred to the information provided in the patient's medical records. Completed survey sheets were anonymized, sent to the lead author by mail, and then analyzed.

This study was conducted after approval by the ethics committee of the National Center of Neurology and Psychiatry (approval number A2019‐060), the principal study facility, and subsequently by the ethics committees of the other eight psychiatric hospitals.

### 2.3 Examined items

As examined items, in addition to general characteristics such as age, biological sex, academic background, and current employment and occupational status (employed or unemployed), items related to the following two areas were assessed:

#### 2.3.1 Psychiatric items

- Clinical genetic factors: Family history of psychiatric disorders, substance dependence/addiction behavior, and suicidal behavior (suicide attempts and completed suicide). Subjects were asked about their second‐degree family history, and “suspected” cases with no treatment history were considered to indicate a family history.
- ICD‐10 diagnostic subclassification “F12—Mental and behavioral disorders due to use of cannabinoids” at the time of the survey
- Diagnostic classification of comorbid psychiatric disorders according to the main ICD‐10 category at the time of the survey, and the temporal relationship between onset and cannabis use.

#### 2.3.2 Items related to the history of cannabis use

- Age at the time of first cannabis use
- Age at the time of final use: In this study, the number of years of cannabis use was calculated automatically as follows: Age at the time of final use minus age at the time of first use. When studying drug dependence, the duration of illicit drug use can be difficult to calculate because drug use is often irregularly interrupted by detention or imprisonment, and self‐reported information can be unreliable. Some studies neglected duration and focused instead on the age when drug use started,^11^, ^13^ while others simply counted the duration using the above method.^39^ In the present study, we adopted this method in addition to collecting the age at which drug use started.
- Cannabis products used (four categories: dried [herbal] cannabis, cannabis resin, liquid cannabis, and other): We examined whether the subject had used cannabis products that contain high levels of THC such as cannabis resin and liquid cannabis. We adopted this dichotomic classification system based on previous findings. Particularly, a seminal study using gas chromatography demonstrated that THC content was highest in liquid products and lowest in herbal products.^40^ Further, a recent study conducted in Japan found that resin products termed “cannabis wax” contained 50 times more THC than general dry cannabis.^41^ These findings suggest that resin and liquid products may contain relatively high levels of THC compared with dried/herbal products.
- Frequency of cannabis use at the time of most frequent use. Only one of the following four categories was selected: Less than once per month, once per month to <1 day per week, 1 day per week to <4 days per week, ≥4 days per week.
- History of combined use of other psychoactive substances during the period of cannabis use (alcohol, methamphetamine [MAP], inhalants, cocaine, opioids, hallucinogens [eg, LSD, MDMA], new psychoactive substances [NPS], hypnotics and anxiolytics, over‐the‐counter [OTC] drugs, and others).

### 2.4 Statistical analysis

Based on the collected information, we conducted binomial logistic regression analysis of all target subjects using two ICD‐10 diagnoses (current “dependence syndrome,” and “residual and late‐onset psychotic disorder,” which refers to a state of chronic psychosis caused by cannabis use) as dependent variables, and then examined the factors associated with these two diagnoses. We selected the following as independent variables: “family history of psychiatric disorders, substance dependence/addiction behavior, and suicidal behavior,” “onset of comorbid psychiatric disorder before starting to use cannabis,” “age at the time of first cannabis use,” “number of years of cannabis use,” “use of cannabis products other than dried cannabis,” and “combined use of other psychoactive substances,” which were identified as risk factors for the onset of dependence and chronic psychosis due to cannabis use in previous studies. We then conducted univariate and multivariate analysis of each of these variables.

All statistical analysis was conducted using SPSS ver. 26 (IBM), and the significance level was set at <5%.

## 3 RESULTS

A total of 73 patients who underwent treatment at the nine mental health hospitals during the survey period fell under the category ICD‐10 “Mental and behavioral disorders due to use of cannabinoids,” and 71 of these patients provided consent to participate in this study (consent rate: 97.3%).

Table 1 shows the subject characteristics. The mean age was 35.1 years (standard deviation [SD] 10.2 years), the mean age at the time of first examination at all mental health hospitals was 31.5 years (SD 8.7 years), and the mean duration of treatment was 3.6 years (SD 4.6 years).

**Table 1:** Subject profile

		Mean age/number of years	SD
Current age (years)		35.1	10.2
Age at the time of initial examination (years)		31.5	8.7
Treatment duration at the study facility (years)		3.6	4.6

		Number of subjects	%
Biological sex	Male	59	83.1
	Female	12	16.9
Educational background	Did not graduate from high school	29	40.8
	Completed high school or higher levels of education	42	59.2
Current employment status	Employed	35	49.3
	Unemployed	36	50.7
Clinical genetic factors	Family history of psychiatric disorders	17	23.9
	Family history of substance dependence/addiction behavior	12	16.9
	Family history of suicidal behavior	4	5.6
Current F12 diagnostic subclassification	(F12.0) Acute intoxication	1	1.4
	(F12.1) Harmful use	7	9.9
	(F12.2) Dependence syndrome	41	57.7
	(F12.3) Withdrawal state	0	0.0
	(F12.4) Withdrawal state with delirium	0	0.0
	(F12.5) Psychotic disorder	8	11.3
	(F12.6) Amnesic syndrome	1	1.4
	(F12.7) Residual and late‐onset psychotic disorder	17	23.9
	(F12.8) Other mental and behavioral disorders	0	0.0
Current presence of comorbid psychiatric disorder	Any of the comorbid psychiatric disorders below	31	43.7
	F0: Organic, including symptomatic, mental disorders	1	1.4
	F2: Schizophrenia, schizotypal and delusional disorders	8	11.3
	F3: Mood (affective) disorders	13	18.3
	F4: Neurotic, stress‐related and somatoform disorders	2	2.8
	F5: Behavioral syndromes associated with physiological disturbance and physical factors	3	4.2
	F6: Disorders of adult personality and behavior	6	8.5
	F7: Mental retardation	1	1.4
	F8: Disorders of psychological development	4	5.6
	F9: Behavioral and emotional disorders with onset usually occurring in childhood and adolescence	2	2.8
	Time of onset of comorbid psychiatric disorder		
	Onset before starting to use cannabis	16	22.5
	Onset after starting to use cannabis	15	21.1

Regarding the biological sex of the 71 subjects, 59 subjects (83.1%) were male, and 12 subjects (16.9%) were female. Regarding educational background, 29 subjects (40.8%) had not graduated from high school, and 42 subjects (59.2%) had completed high school or higher levels of education. Regarding the employment status at the time when this survey was conducted, 35 subjects (49.3%) had some type of job, and 36 subjects (50.7%) were unemployed.

Regarding clinical genetic factors, 17 subjects (23.9%) had a family history of psychiatric disorders, 12 subjects (16.9%) had a family history of substance dependence/addiction behavior, and four subjects (5.6%) had a family history of suicidal behavior.

Regarding the ICD‐10 F12 diagnostic subclassification of subjects, 41 subjects (57.7%) fell under the category “(F12.2) Dependence syndrome,” which was the most common, followed by 17 subjects (23.9%) who fell under the category “(F12.7) Residual and late‐onset psychotic disorder.” This was followed by “(F12.5) Psychotic disorder” in eight subjects (11.3%), “(F12.1) Harmful use” in seven subjects (9.9%), and “(F12.0) Acute intoxication” and “(F12.6) Amnesic syndrome” in one subject (1.4%) each. No subject fell under the categories “(F12.3) Withdrawal state,” “(F12.4) Withdrawal state with delirium,” or “(F12.8) Other mental and behavioral disorders.”

Regarding comorbid psychiatric disorders confirmed at the time of this survey, 31 subjects (43.7%) were confirmed to have some sort of comorbid psychiatric disorder. The most common was “F3: Mood disorder,” which was identified in 13 subjects (18.3%), followed by “F2: Schizophrenia, schizotypal, and delusional disorders” in eight subjects (11.3%), “F6: Disorders of adult personality and behavior” in six subjects (8.5%), “F8: Disorders of psychological development” in four subjects (5.6%), “F5: Behavioral syndromes associated with physiological disturbance and physical factors” in three subjects (4.2%), “F4: Neurotic, stress‐related, and somatoform disorders” and “F9: Behavioral and emotional disorders with onset usually occurring in childhood and adolescence” in two subjects (2.8%) each, and “F0: Organic, including symptomatic, mental disorders” and “F7: Mental retardation” in one subject (1.4%) each. Regarding the earliest onset of comorbid psychiatric disorders, onset in 16 subjects (22.5%) occurred before they started to use cannabis.

Table 2 provides information regarding the subjects' modes of cannabis use. The mean age at the time of first cannabis use was 19.5 years (SD 4.0 years), and the mean number of years of cannabis use (calculated from the difference between the age at the time of final use and the age at the time of first use) was 12.8 years (SD 8.7 years).

**Table 2:** Subjects' modes of cannabis use

		Mean age/number of years	SD
Age at the time of first cannabis use (years)		19.5	4.0
Number of years of cannabis use (years)		12.8	8.7

		Number of subjects	%
Types of cannabis products used	Use of cannabis products other than dried cannabis	36	50.7
	Dried cannabis	70	98.6
	Cannabis resin	36	50.7
	Liquid cannabis	14	19.7
Frequency of cannabis use at the time of most frequent use	Less than once a month	2	2.8
	Once a month to <1 d a week	4	5.6
	1 d a week to <4 d a week	20	28.2
	≥4 d a week	45	63.4
Habitual use of other psychoactive substances during the period of cannabis use	Used either of the psychoactive substances	55	77.5
	Alcohol	25	35.2
	MAP	26	36.6
	Inhalants	16	22.5
	Cocaine	16	22.5
	Opioids	2	2.8
	Hallucinogens (eg, LSD, MDMA)	23	32.4
	NPS	22	31.0
	Hypnotics, anxiolytics	11	15.5
	OTC drugs	4	5.6
	Others	0	0.0

Abbreviations: MAP, Methamphetamine; NPS, New psychoactive substances; OTC drugs, over‐the‐counter drugs.

Regarding the cannabis products that had been used, almost all subjects (70 subjects [98.6%]) had used dried cannabis, 36 subjects (50.7%) had used cannabis resin, 14 subjects (19.7%) had used liquid cannabis, and 36 subjects (50.7%) had some experience with using cannabis products other than dried cannabis.

Regarding the rate of usage in the period in which cannabis use was most frequent, “≥4 days per week” was the most common response, given by 45 subjects (63.4%), followed by “1 day per week to <4 days per week” in 20 subjects (28.2%), “once per month to <1 day per week” in four subjects (5.6%), and “less than once per month” in two subjects (2.8%).

Concerning the habitual use of other psychoactive substances during the period of cannabis use, 55 subjects (77.5%) reported using one additional psychoactive substance. Regarding the type of psychoactive substance, the most common was “alcohol,” reported in 25 subjects (35.2%), followed by “MAP” in 26 subjects (36.6%), “hallucinogens” in 23 subjects (32.4%), “NPS” in 22 subjects (31.0%), “inhalants” and “cocaine” in 16 subjects (22.5%) each, “hypnotics, anxiolytics” in 11 subjects (15.5%), “OTC drugs” in four subjects (5.6%), and “opioids” in two subjects (2.8%).

Table 3 shows the results of the logistic regression analysis regarding the current diagnosis of “F12.2 Dependence syndrome due to use of cannabinoids.” Univariate analysis revealed that the “number of years of cannabis use” (15.6 years [SD 7.7 years] vs. 9.0 years [SD 8.7 years], *P* = .003, odds ratio [OR] 1.109, 95% confidence interval [CI; 1.036‐1.187]), “use of cannabis products other than dried cannabis” (80.6% vs. 23.3%, *P* < .001, OR 7.940, 95% CI [2.694‐23.404]), and “usage for ≥4 days per week” (75.6% vs. 46.7%, *P* = .014, OR 3.543, 95% CI [1.289‐9.739]) were significantly associated factors. However, the multivariate analysis extracted only the “number of years of cannabis use” (*P* = .020, OR 1.094, 95% CI [1.014‐1.180]) and “use of cannabis products other than dried cannabis” (*P* = .004, OR 6.850, 95% CI [1.866‐25.145]) as significantly associated factors.

**Table 3:** Logistic regression analysis regarding the current diagnosis of “F12.2 Dependence syndrome due to use of cannabinoids”

	(F12.2) Dependence syndrome				Univariate analysis						Multivariate analysis					
	Applies		Does not apply		B	Wald	*P*	Odds ratio	95% CI		B	Wald	*P*	Odds ratio	95% CI	
	N = 41		N = 30						Lower	Upper					Lower	Upper
Family history of psychiatric disorders	9	22.0%	8	26.7%	0.257	0.211	.646	1.293	0.432	3.869	0.192	0.061	.805	1.212	0.263	5.579
Family history of substance dependence/addiction behavior	5	12.2%	7	23.3%	0.784	1.486	.223	2.191	0.621	7.735	1.400	2.519	.113	4.054	0.720	22.842
Family history of suicidal behavior	2	4.9%	2	6.7%	0.331	0.103	.748	1.393	0.185	10.491	−0.844	0.352	.553	0.430	0.027	6.968
Onset of comorbid psychiatric disorder before starting to use cannabis	7	43.8%	9	30.0%	−0.733	1.624	.203	0.480	0.156	1.484	−0.874	1.414	.234	0.417	0.099	1.763
Age at the time of first cannabis use (years)	19.2	SD 4.3	19.8	SD 3.6	−0.032	0.282	.595	0.968	0.860	1.090	0.069	0.594	.441	1.072	0.899	1.277
Number of years of cannabis use (years)	15.6	SD 7.7	9.0	SD 8.7	0.103	8.951	.003	1.109	1.036	1.187	0.090	5.422	.020	1.094	1.014	1.180
Use of cannabis products other than dried cannabis	29	80.6%	7	23.3%	2.072	14.115	<.001	7.940	2.694	23.404	1.924	8.410	.004	6.850	1.866	25.145
≥4 d a week	31	75.6%	14	46.7%	1.265	6.011	.014	3.543	1.289	9.739	0.743	1.164	.281	2.102	0.545	8.109
Combined use of other psychoactive substances	34	82.9%	21	70.0%	0.733	1.624	.203	2.082	0.674	6.429	0.566	0.601	.438	1.761	0.421	7.366

Note

Univariate/multivariate logistic regressions: Applies = 1, Does not apply = 0.

Table 4 shows the results of the logistic regression analysis regarding the current diagnosis of “F12.7 Residual and late‐onset psychotic disorder due to use of cannabinoids.” Univariate analysis revealed that only “use of cannabis products other than dried cannabis” was a significantly associated factor (23.5% vs. 59.3%, *P* = .014, OR 0.212, 95% CI [0.061‐0.735]). However, multivariate analysis extracted two factors, “onset of comorbid psychiatric disorder before starting to use cannabis” (5.9% vs. 27.8%, *P* = .049, OR 0.104, 95% CI [0.011‐0.987]) and “use of cannabis products other than dried cannabis” (*P* = .014, OR 0.212, 95% CI [0.061‐0.735]) as significantly associated factors.

**Table 4:** Logistic regression analysis regarding the current diagnosis of “F12.7 Residual and late‐onset psychotic disorder due to use of cannabinoids"

	(F12.7) Residual and late‐onset psychotic disorder				Univariate analysis						Multivariate analysis					
	Applies		Does not apply		B	Wald	*P*	Odds ratio	95% CI		B	Wald	*P*	Odds ratio	95% CI	
	N = 17		N = 54						Lower	Upper					Lower	Upper
Family history of psychiatric disorders	5	29.4%	12	22.2%	0.377	0.365	.546	0.686	0.201	2.334	0.520	0.333	.564	1.681	0.288	9.809
Family history of substance dependence/addiction behavior	3	17.6%	9	16.7%	−0.069	0.009	.925	0.933	0.222	3.930	0.606	0.376	.540	1.832	0.264	12.699
Family history of suicidal behavior	2	11.8%	2	3.7%	1.243	1.423	.233	0.288	0.037	2.224	−1.493	0.714	.398	0.225	0.007	7.167
Onset of comorbid psychiatric disorder before starting to use cannabis	1	5.9%	15	27.8%	−1.817	2.859	.091	0.163	0.020	1.225	−2.264	3.885	.049	0.104	0.011	0.987
Age at the time of first cannabis use (years)	19.2	SD 3.6	19.6	SD 4.1	−0.029	0.155	.694	0.972	0.842	1.121	−0.084	0.731	.392	0.919	0.758	1.115
Number of years of cannabis use (years)	11.2	SD 8.6	13.3	SD 8.7	−0.029	0.730	.393	0.972	0.909	1.038	−0.007	0.029	.865	0.993	0.914	1.079
Use of cannabis products other than dried cannabis	4	23.5%	32	59.3%	−1.553	5.978	.014	0.212	0.061	0.735	−2.171	7.219	.007	0.114	0.023	0.556
≥4 d a week	10	58.8%	35	64.8%	−0.254	0.199	.655	0.776	0.254	2.367	0.162	0.046	.831	1.176	0.265	5.217
Combined use of other psychoactive substances	15	88.2%	40	74.1%	0.965	1.405	.236	2.625	0.532	12.950	1.608	2.806	.094	4.991	0.761	32.738

Note

Univariate/multivariate logistic regressions: Applies = 1, Does not apply = 0.

## 4 DISCUSSION

To the best of our knowledge, the current study examined the largest sample of patients with cannabis‐related psychiatric disorders in Japan. Moreover, to date, this is the only study in Japan to examine risk factors for the onset of dependence and chronic psychosis due to cannabis use by considering of the impact of clinical genetic factors, preceding psychiatric disorders, and the mode of cannabis use.

As factors associated with the onset of dependence due to cannabis use, the univariate analysis identified the number of years of cannabis use, the use of cannabis other than dried cannabis, and usage on ≥4 days per week, while the multivariate analysis identified the number of years of cannabis use and the use of cannabis products other than dried cannabis. These findings are consistent with those of previous studies by Coffey et al,^6^ Grant and Pickering,^7^ and Noack et al^8^ Moreover, the current results suggest that cannabis use for a longer period and at a higher frequency, as well as intake of products with a high THC content, may have a larger impact on the onset of dependence than clinical genetic factors, the combined use of other psychoactive substances, or the presence of psychological distress before starting to use cannabis. In other words, as with alcohol,^42^ a higher frequency and longer period of THC intake, as well as a higher concentration of THC intake (ie, the total intake of THC), are associated with a higher risk of developing dependence.

We obtained unexpected results regarding the onset of residual and late‐onset psychotic disorders. In this study, as factors associated with the onset of these disorders, the univariate analysis identified the use of cannabis other than dried cannabis, while the multivariate analysis identified the presence of a comorbid psychiatric disorder before starting to use cannabis and the use of cannabis other than dried cannabis, with ORs below the decimal point (=negative association). This finding suggests the possibility that the duration and frequency of cannabis use, early start of cannabis use, and clinical genetic factors may not be associated with the onset of chronic psychosis due to cannabis use. Rather, the associated factors were (a) no prior history of use of cannabis products with high THC levels and (b) no history of psychiatric disorders prior to cannabis use.

This result is not consistent with previous studies that identified risk factors for the onset of chronic psychosis, including genetic factors,^28^, ^29^ cannabis use early in life,^13^ the use of cannabis products with a high THC content,^30^ and cannabis use to alleviate symptoms caused by psychiatric disorders.^31^ Further, the current results contradict the findings of several previous studies. Particularly, our finding of a negative association between chronic psychosis due to cannabis and the presence of a comorbid psychiatric disorder prior to cannabis use is contrary to the reported association between chronic psychosis due to cannabis and the use of cannabis for alleviating pre‐existing psychiatric disorders.^31^ Further, our finding of a negative association between chronic psychosis due to cannabis and the use of cannabis products other than dried cannabis (resins and liquids with a high THC content) is contrary to the reported association between chronic psychosis due to cannabis and the use of cannabis products with a high THC content.^30^

The present data reveal an association between the absence of comorbid psychiatric disorders prior to cannabis use and chronic psychosis due to cannabis use. This finding could have resulted from several factors. In the case of patients who had some type of psychiatric disorder before starting to use cannabis, chronic psychosis was not considered to be after effect of cannabis use (residual and late‐onset psychotic disorders), but instead, as a disease state in which latent schizophrenia was induced by cannabis use, and thus was counted as a comorbid psychiatric disorder. In that sense, the finding that only a small number of patients who were diagnosed with residual and late‐onset psychotic disorders had comorbid psychiatric disorders before starting to use cannabis was an expected result given the definition of the diagnostic criteria and disease state.

In contrast, it is difficult to explain the observed association between chronic psychosis due to cannabis use and no history of using cannabis products with a high THC content. A mentioned above, the current findings indicate that the frequency and duration of exposure to THC—the constituent responsible for the psychotropic activity of cannabis—were not associated with the onset of chronic psychosis. In that context, it is unsurprising that the use of cannabis products with a high THC content was not related to the onset of chronic psychosis. These suggest that the onset of cannabis‐related chronic psychosis may be more influenced by individual/constitutional factors, including clinical genetic factors and those that precede the onset of comorbid psychiatric disorders, than the total amount of THC exposure, although we found no associations between individual/constitutional factors and chronic psychosis in this study.

The reason why a significant negative association was observed between the use of high THC‐containing products and chronic psychosis is not clear. Two possibilities may be involved in the above result. One possibility is the heterogeneity of cannabis‐related chronic psychosis in which not only pure psychosis but also other disorders such as organic personality disorder, organic mood disorder, and dementia are included. The other possibility is the small sample size of subjects which may have influenced the statistical output. Therefore, further investigation should be required in this respect.

The current study has several limitations that should be considered. First, the “number of years of cannabis use” was automatically calculated from the difference between the “age at the time of final cannabis use” and the “age at the time of first cannabis use.” Periods of voluntary drug abstinence and periods of forced drug abstinence while serving a prison term were not considered. Therefore, the number of years of cannabis use and the total amount of cannabis used may not be positively correlated. Second, the ICD‐10 “Residual disorders and late‐onset psychotic disorder” is a heterogenic group including rare psychiatric cannabis‐related conditions in clinical practice, such as flashbacks, organic personality disorder, organic mood disorder, and dementia, in addition to chronic psychosis. Therefore, the risk factors identified in this study may be inconsistent with those of purely chronic psychosis. Third, some cases of chronic psychosis due to cannabis use may not have been listed as “residual and chronic late‐onset psychotic disorder,” but could have been listed as a comorbid psychiatric disorder, namely, cannabis‐induced schizophrenia. Forth, because this study was conducted in patients who were undergoing treatment at a psychiatric hospital, the sample may not have been representative of cannabis users in the general population. In our sample, 43.7% of all subjects had some sort of comorbid psychiatric disorder at the time when the survey was conducted. Of this subset, approximately half (22.5% of all subjects [16 of 71 subjects]) had been suffering from some type of psychiatric disorder before they started to use cannabis. Thus, because the subjects in the current study had various psychiatric problems before they started to use cannabis, they were likely to have different characteristics compared with cannabis users in the general population. Fifth, the sample size was too small for in‐depth statistical examination, and the possibility of type II errors cannot be excluded. Sixth, because all attending psychiatrists used an interview‐based method in which they asked patients questions about the mode of cannabis use in the past, it was difficult to avoid recall bias. Finally, in this study, we collected information from several attending psychiatrists at nine mental health hospitals, and the evaluation criteria might have varied between institutions. It should be emphasized, however, that each of these nine mental health hospitals treat many patients with drug dependence daily, and all attending psychiatrists had a high level of expertise for diagnosing and treating drug dependence.

Despite the limitations described above, the current study represents an important contribution as the only study of its kind in Japan. We examined the largest sample of patients to date with cannabis‐related psychiatric disorders in Japan to investigate the onset of dependence and chronic psychosis due to cannabis use while considering clinical genetic factors, preceding psychiatric issues, and the mode of cannabis use.

The current study examined risk factors for the onset of dependence and chronic psychosis due to cannabis use in 71 patients with cannabis‐related psychiatric disorders who underwent treatment at nine mental health hospitals in Japan. The results suggested that the onset of dependence may be related to long‐term cannabis use and the use of cannabis products with a high THC content. However, regarding the onset of chronic psychosis, no association was observed for total intake of THC (including duration and frequency of use), or for psychiatric vulnerability (including clinical genetic factors and the presence of preceding psychiatric disorders). These findings indicate that the onset of chronic psychosis is related to unknown factors.

## CONFLICT OF INTEREST

We declare that we have no conflicts of interest in relation to this study.

## AUTHOR CONTRIBUTIONS

TM, TK, and TS designed the study, and TM drafted the main manuscript. TM, DF, TU, and MM analyzed the data, and KO and YT reviewed previous studies. All the authors collected data and drafted tables and parts of the manuscript.

## ETHICAL APPROVAL

Approval of the research protocol by an Institutional Reviewer Board: This study was conducted after approval by the ethics committee of the National Center of Neurology and Psychiatry (approval number A2019‐060), the principal study facility, and subsequently by the ethics committees of the other eight psychiatric hospitals.

## INFORMED CONSENT

In addition to giving public notice regarding the implementation of the study, the candidate was given a face‐to‐face explanation of the study and verbal consent was obtained.

## Data Availability

The data that support the findings of this study are available on request from the corresponding author. The data are not publicly available due to privacy or ethical restrictions.
